# Practical methods for incorporating summary time-to-event data into meta-analysis: updated guidance

**DOI:** 10.1186/s13643-025-02752-z

**Published:** 2025-04-10

**Authors:** Jayne F. Tierney, Sarah Burdett, David J. Fisher

**Affiliations:** MRC Clinical Trials Unit, Medical Research Council Clinical Trials Unit, University College London, London, UK

**Keywords:** Meta-analysis, Methods, Time-to-event outcomes, Hazard ratio, Summary data, Aggregate data

## Abstract

Our previous guide to estimating hazard ratios (HRs) from published summary (aggregate) data has become very widely used, but many still have difficulties knowing when and how to apply the methods. Informed by our increased experience of applying them across a range of settings, the queries we have received and results of a survey of Cochrane editors on the methods, we have updated the guidance comprehensively. Previously, we described a range of scenarios for deriving a HR and logrank variance (V) from published time-to-event analyses. They are incorporated in this update, together with clarification of ambiguities and additional scenarios. We also provide further guidance on extracting and using data from publications and Kaplan–Meier (KM) curves, raise some of the challenges, and discuss recent alternatives to the “Parmar” KM methods. A new calculations spreadsheet will perform all possible calculations given the data that are entered and includes new features to enhance the user experience. This updated guidance and associated spreadsheet represent valuable additional tools for those conducting meta-analyses based on published, summary, time-to-event data.

**Supplementary Information:**

The online version contains supplementary material available at 10.1186/s13643-025-02752-z.

## Background

Our previous guide to estimating hazard ratios (HRs) from published summary (aggregate) data [1] has become very widely used, but many still have difficulties knowing when and how to apply these methods. HRs are useful for exploring the effects of treatments on time-to-event outcomes. These are defined by both the number and the timing of events, such as time to disease progression or to the relief of symptoms, or time to last follow-up for participants not experiencing an event (i.e., that have been censored). Ideally for meta-analysis of such outcomes, an HR and some measure of the associated variance would be extracted directly from a trial report. Nowadays, trial reports often include a log HR and the associated standard error (SE), and these can be used directly in meta-analysis [2]. Similarly, if an HR and confidence interval are reported, it is easy to compute a log HR and SE from these [2], including via the spreadsheet accompanying this article.

For trial reports that are old, brief, or do not conform to modern reporting standards, such statistics may not be provided. Therefore, unless the necessary statistics can be obtained from trial investigators or derived from individual participant data (IPD), researchers must either calculate or estimate an HR and its variance from other published statistics or data extracted from Kaplan–Meier (KM) curves. Although other papers had described how to do this [3, 4], the methods were challenging for researchers with limited statistical training. Hence, we created our previous guide to explain the methods in simpler terms and according to which published statistics and/or data are available, with an accompanying spreadsheet to facilitate the necessary calculations [1].

The guide became the Trials Journal’s most-cited paper of all time, being cited over 5000 times (Source: www.scopus.com, January 2025). While this demonstrates extensive use by systematic reviewers and meta-analysts worldwide, our experience across a range of settings, the queries we have received, and the results of a survey of Cochrane editors [5] show that gaps and misconceptions prevail. Among the most problematic aspects identified by the survey relate to the assumptions underpinning the analytic methods, reconstruction of data from primary reports, and interpretation of effects, with additional issues including proportionality of hazards, competing events, censoring, and absolute effects [5]. Moreover, alternative methodologies have since emerged, hence the need for a comprehensive update of the guidance, with additional tips and a new calculations spreadsheet.

## Methods

### The basis of the methods

The hazard ratio (HR) is typically the most appropriate measure for summarizing the effect of an intervention on a time-to-event outcome [3]. For the purposes of the current article, we define the hazard within each arm of a trial as the ratio of the number of events observed to the number expected, had events occurred equally across the two arms [6]. This leads to the following “direct” expression for a HR:

1 $$\mathrm{HR}=\left[\frac{\text{Observed events research} / \text{Expected events research}}{\text{Observed events contro1}/ \text{Expected events control}}\right]$$

Note that in this equation, and throughout, we use “research” to denote the research intervention arm (or group) and “control” to denote the standard or control arm (or group).

However, it is now commonplace for a HR to be estimated using Cox proportional-hazards regression. The coefficient for the effect of the research treatment from such a model provides an alternative “direct” estimate of the log HR [7], with an associated standard error (SE) and *p* value. Such log HRs and SEs can be entered into standard meta-analysis software such as Cochrane’s Revman [8] or comprehensive meta-analysis [9], or be used with meta-analysis packages within Stata [10] and R [11].

The logrank test [12] is a commonly used test for comparing KM curves. The test statistic uses the squared difference between observed and expected events in the research arm (*O-E*), divided by the “logrank” or “hypergeometric” variance (*V*) [13]:

2 $$\text{Logrank test statistic }\sim {\chi }^{2}\text{ on 1 d.f.}=\frac{{\left({\mathrm{Observed}}-\text{Expected events research}\right)}^{2}}{\text{Logrank variance}}$$

As the *p* values and inference from this and from Cox regression are approximately equal [14], the logrank statistic may be used to obtain a HR indirectly (often referred to as the “Peto HR” [15]), with “exp” denoting the exponential or anti-log function:

3 $${\mathrm{HR}}=\mathrm{exp}\left(\frac{{\mathrm{Observed}}-\text{Expected events research}}{\text{Logrank variance}}\right)$$

**Fig. 1:**
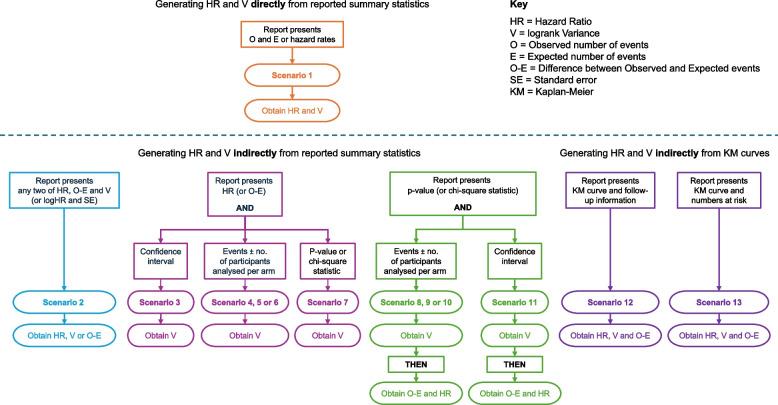
Overview of scenarios for estimating hazard ratios from published time-to-event data

The guidance presented here and in previous papers [3, 4] aims to show how direct and indirect methods can be used to estimate a HR and associated *V* from available published information, with the range of scenarios summarized in Fig. 1.

The more indirect the method, the stronger the assumptions required. Therefore, direct methods are always preferable, followed by indirect methods based on reported statistics, and then those based on KM curves [1]. If reported data allows, estimation using multiple methods can be achieved easily using the accompanying calculations spreadsheet, providing a sensitivity analysis across methods and a useful check of any statistics supplied by investigators.

### What this update provides

We provide a range of scenarios for deriving a HR and *V,* some described previously [1], plus some new additions (summarized in Fig. 1), and the user may select those most suited to their needs. Scenario 1 assumes that HR and *V* are directly available (or may be derived directly using observed and expected event counts). Scenarios 2 to 6 assume that a HR or *O-E* for the research arm is available and provide different ways of approximating *V* from other published information (Fig. 1). Scenarios 7 to 11 assume that only a *p* value or test statistic is available, but with sufficient additional information to approximate *V*, which then enables derivation of an approximate *O-E* and HR (Fig. 1). Finally, Scenarios 12 and 13 make use of data extracted from published KM curves to estimate a HR and *V*, using a modified life-table approach [1]. All the indirect methods of estimating a HR and *V* might be considered as a form of imputation and may not be appropriate if the proportional hazards assumption is violated [7].

The layout and numbering of scenarios have been kept as close as possible to the original paper, with specific updates to the spreadsheet noted where necessary. We have adopted a similar nomenclature and simplified approach to the equations so that both articles may be used in tandem. All equations have been arranged in terms of a HR and *V,* and words and phrases are used within the equations so that they can be easily understood (except the quantities HR, *V*, *O-E* and SE). 

The numbers analyzed and the corresponding numbers of events entered in the equations should be those used to produce the reported result or KM curve. Often, these will represent all participants randomized, or instead, a subset of those randomized (e.g., if some were excluded for a per-protocol analysis or due to post-randomization eligibility checking), or a participant subgroup of interest (e.g., women).

Additional file 1 summarizes the methods using the same equation numbering as in the appendix of [1] but uses formal mathematical notation and provides derivations from first principles. A similar summary and derivation of the “Parmar” methods is given in Additional file 2. The updated spreadsheet, in Additional file 3, will perform all possible calculations given the data that are entered and includes new features to enhance the user experience. A brief user guide for the spreadsheet is provided in Additional file 4.

To illustrate the methods, we use the same examples as used previously [1]: one relating to an ovarian cancer trial [16] and another to a bladder cancer trial [17]. Both evaluated the effects of chemotherapy versus no chemotherapy on the outcome of overall survival. Note that, in the worked examples, numbers have been rounded to two decimal places for presentation, but not for the underlying calculations.

Throughout, we clarify ambiguities and provide further tips on extracting and using appropriate data from publications and from KM curves. We briefly discuss some of the challenges of using reported time-to-event analyses, such as choosing between adjusted and unadjusted HRs or dealing with the presence of non-proportional hazards, and discuss some recent alternatives to the Parmar KM curve methods [3]. It should be noted, however, that the guidance cannot rectify issues that arise from the design or analysis of individual trials.

## Generating the HR and V from reported summary statistics

At the outset, it is worthwhile extracting all the necessary descriptive and statistical information for each outcome of interest and for each trial using a standard form, as we have done for the bladder cancer example (Table 1).

**Table 1:** Data extracted from the report of the example trial in bladder cancer [17] for the outcome of survival (adapted with permission from [1])

Trial reference: *BA06*	Research (chemotherapy)	Control (no chemotherapy)
Randomisation ratio (e.g., 1:1)	1	1
Participants randomized	491	485
Participants analyzed	491	485
Observed events	229	256
Logrank expected events	Not reported	Not reported
HR and CI (level, e.g., 95%)	0.85, 95% CI 0.71 to1.02	
Logrank variance	Not reported	
Logrank observed minus-expected events	Not reported	
Test statistic	Not reported	
Two-sided *p*-value to two significant figures (test used, e.g., logrank, Mantel-Haenzsel or Cox)	0.075, from logrank test	
Advantage to research or control?	Research	
HR and CI (level, e.g., 95%) or SE or V from adjusted or unadjusted Cox model	Not reported	
Kaplan–Meier, actuarial or cumulative incidence curves reported?	Yes, Kaplan–Meier	
Numbers at risk reported	Yes, at yearly intervals to 5 years	
Accrual period	11/89 to 7/95 (69 months)	
Median, minimum, and maximum follow-up	Median = 48 months Min = 14 months Max = 82 months (Min and Max were estimated from the accrual period and median follow-up as per Additional file 2)	

### 1. Report presents observed and expected events for research and control arms

If the observed and (logrank) expected events for the research and control arms are presented in a trial report, then a HR can be calculated directly using Eq. 1, as described above, with *V* calculated using Eq. 4:

4 $$V=\frac{1}{\left(1/\text{Expected events research}\right)+\left(1/\text{Expected events control}\right)}$$

As these quantities were reported for the ovarian cancer trial [16], they can be used to obtain a direct estimate of the HR of 1.51 and *V* of 14.46:

Observed events, research = 34 Expected events, research = 28.0

Observed events, control = 24 Expected events, control = 29.9

$${\mathrm{HR}}=\frac{34/28.0}{24/29.9}=1.51\;V=\frac{1}{\left[\left(1/28.0\right)+\left(1/29.9\right)\right]}=14.46$$

Note that the total expected events given in the trial report are 57.9 rather than the 58 observed [16], triggering an error message in the spreadsheet. As the discrepancy is small and likely due to rounding, in this case, the message may safely be ignored.

If the hazard rates (i.e., the ratio of observed to expected events) for the research and control arms are included in a trial report, they can replace the top and bottom of Eq. 1, instead of the observed and expected events. To exemplify, the data above would produce a hazard rate for the research arm of 1.21 and control arm of 0.80 and the same HR of 1.51. However, as hazard rates cannot be used to calculate *V*, this would need to be estimated using one of the “indirect” methods in the following scenarios.

### 2. Report presents any two of HR, O-E, and logrank V (or logHR and standard error)

If a trial report presents any two of the HR, *V*, and *O-E* events for the research arm, the missing statistic can be calculated directly from Eq. 2 or its re-arrangements below:

5 $$V=\frac{O-E}{\text{log HR}}$$

6 $$O-E=\left({\mathrm{log}}\;{\mathrm{HR}}\right)\times V$$

These equations are used in many subsequent scenarios, for example, in Scenarios 8 to 11 to obtain a HR from a *O-E* and *V*. HRs calculated in this way will not differ markedly from those obtained using the formal definition in Scenario 1, unless the event rate in a trial is low [3].

For illustration purposes, the data derived from the ovarian cancer trial report [16] and Scenario 1 are used in Eq. 3 to give a HR of 1.51:

$$\begin{array}{c}\begin{array}{cc}O-E=34-28=6.00& V=14.46\end{array}\\ {\mathrm{HR}}={\mathrm{exp}}\left[\frac{6.00}{14.46}\right]= 1.51\end{array}$$

As described above, it is now common for trials to report a log HR and its SE. If a HR and *V* are required for presentation or other purposes, the former is simply the exponential of the log HR, and Eq. 7 shows how *V* relates to the SE. While our previous guidance [1] also referred to the variance of the log HR (denoted V*), which is the reciprocal of the logrank variance, *V,* and equal to the square of the standard error, here, we use only the SE to minimize confusion.

7 $$V=\frac{1}{{\left[\text{SE of log HR}\right]}^{2}}$$

- ➢ The updated version of the spreadsheet (Additional file 3) will generate all possible estimates of the HR and *V*, and of the log HR and its standard error, from the quantities entered.

### 3. Report presents HR (or O-E) and confidence interval

When a HR and associated confidence interval (CI) are presented in a trial report, *V* can be estimated from the CI, provided it is given to at least two significant figures [3, 4]:

8 $$V = \, {\left[\frac{2\times \left(\text{critical value for CI}\right)}{{\text{log (Upper CI) - log (Lower CI)}}}\right]}^{2}$$

In the usual scenario of a 95% CI being presented, the critical value would be 1.96. The denominator makes use of the natural logarithms of the reported upper and lower confidence limits for the HR. If a different CI is reported, it can still be used in Eq. 8. For example, if a 99% CI is reported, the limits would be entered into the denominator, but the associated critical value in the numerator would be 2.58. Similarly, for a 90% CI the associated critical value would be 1.64.

We demonstrate this and the rest of the indirect methods using data extracted from the bladder cancer trial report [17] (Table 1). Inserting the 95% CI for the HR of 0.71 to 1.02 and the critical value of 1.96 into Eq. 8 gives an estimate of *V* of 117.07:

$$V= {\left[\frac{3.92}{{\mathrm{log}}\left(1.02\right)- {\mathrm{log}}(0.71) \, }\right]}^{2}=117.07$$

### 4. Report presents HR (or O-E) and events in each arm

Where a HR (or *O-E*) is reported with the numbers of events, the latter can be used to estimate *V* via this scenario, or Scenarios 5 or 6. As described above, it is important that the events relate to the sample of participants analyzed and to the associated HR. For completeness, the spreadsheet enables input of both the numbers of participants randomized and analyzed, but calculations are based on the numbers analyzed. If results based on all randomized participants and a subset are both presented (e.g., as “intention to treat” and “per protocol” analyses), it is usually preferable to use the former.

Where a HR (or *O-E*) is reported with the number of events for each arm, a reasonable approximation of *V* may be obtained using Eq. 9.

9 $$V=\frac{\text{Observed events research} \, \times \text{ Observed events control}}{\text{Total events}}$$

Inserting data from the bladder cancer trial gives an estimate of 120.87 for *V*.

$$V=\frac{229\times 256}{485 \, }=120.87$$

Note that it was stated ambiguously in Parmar [3] and incorrectly in Tierney [1] that this scenario also required a 1:1 treatment allocation. In fact, knowledge of the number of events in each arm dispenses with this requirement, and the latest version of the calculations spreadsheet makes this clear.

### 5. Report presents HR (or O-E) and total events (randomization ratio must be 1:1)

If only the total number of events across both arms is reported, then provided the randomization ratio is 1:1, *V* can still be approximated as in Eq. 10:

10 $$V=\frac{\text{Total observed events}}{4}$$

Equation 10 is easily derived from Eq. 11 (below) provided the numbers analyzed on the research and control arms are equal (see Additional file 1). In practice, the numbers on each arm may differ slightly, even with a 1:1 treatment allocation, but the equation may still be used, albeit with a slight loss of accuracy.

Using the total number of events from the bladder cancer trial report gives an estimate of 121.25 for *V*.

$$V=\frac{485}{4}=121.25$$

The simplicity of this method of estimating *V* means it can provide a rapid way to assess the plausibility of estimates of *V* derived using Eqs. 8, 9, or 11.

### 6. Report presents HR (or O-E), total events and the numbers analyzed in each arm

If a HR is reported with a total number of events and the number of participants analyzed in the research and control arms, this allows another means of estimating *V*, irrespective of the treatment allocation ratio (see Additional file 1):

11 $$V=\frac{\text{Total observed events}\times \text{Analyzed research}\times \text{Analyzed control}}{{\left(\text{Analyzed research}+\text{Analyzed control}\right)}^{2}}$$

In the bladder cancer trial example [17], all randomized participants were included in the analysis (Table 1). Using these in Eq. 11, we obtain an estimate of *V* of 121.25.

$$V= \frac{485\times 491\times 485}{{\left(491+485\right)}^{2}}=121.25$$

For a trial designed to have a 1:1 allocation ratio, but which subsequently analyzed notably unequal numbers of participants in each intervention arm (e.g., following a review of eligibility), Eq. 11 is preferable to Eq. 10.

### 7. Report presents HR (or O-E) and p value or chi-square statistic

Our previous guidance [1] did not include methods for using a HR with a *p* value or logrank statistic. As explained above, a logrank test will typically give a very similar *p* value to a Cox regression by treatment. Hence, this and subsequent related Scenarios (7 to 10) are applicable to *p* values associated with either test. The *p* value is back-transformed to obtain a standard normal *z* score, which is used to obtain an estimate of *V*.

Provided the *p* value is reported exactly, and to two or more significant figures [3, 4], Eq. 12 may be used (see Additional file 1):

12 $$V={\left[\frac{\text{z score for }\left({{p}}\text{ value}\div 2\right)}{{\textrm{logHR}}}\right]}^{2}$$

A *p* value of 0.075 was reported for the bladder cancer trial, with an associated *z* score of 1.78. Given this and a HR of 0.85, *V* is estimated as 120.02:

$$V={\left[\frac{1.78}{\mathrm{log}\;0.85}\right]}^{2}=120.02$$

If the test statistic itself is available [3], it will often be a number larger in magnitude, and reported to a greater number of significant figures, than its associated *p* value. Hence, Eq. 13 below is probably a better choice for estimating *V* while also removing the need for back-transformation of the *p* value. However, in the bladder cancer example, the test statistic was not reported.

13 $$V=\frac{\text{chi-square logrank statistic}}{{\left({\textrm{log}}\;{\textrm{HR}}\right)}^{2}}$$

Note that a chi-square test statistic or associated *p* value from a comparison of event rates in the research and control arms does not take censoring into account and is therefore unsuitable for estimating a HR and *V*. Furthermore, it must be emphasized that the use of other test statistics (and their *p* values) typically would not be appropriate. In particular, alternatively weighted logrank tests such as Wilcoxon [18] are designed to detect treatment differences under assumptions other than proportional hazards and therefore may not be consistent with the relevant HR.

- ➢ The updated version of the spreadsheet (Additional file 3) can make use of a HR and either the associated *p* value or chi-squared test statistic to estimate *V*.

### 8. Report presents *p* value (or chi-square statistic) and events in each arm

Where a *p* value (or chi-square statistic) is reported with the numbers of events, this Scenario and Scenarios 9 and 10 make use of a two-step approach to obtain first *V*, then a HR (Fig. 1). If the report presents the events in each arm, *V* can be estimated using Eq. 9, as in Scenario 4:

Then, *V* and the *p* value (or chi-square statistic) can be used together to derive an estimate of the *O-E*:

14 $$O-E=\left(\pm \sqrt{\mathrm{V}}\right)\times {\text{z score for }}({{p}}\text{ value}\div 2)$$

While both Parmar [3] and the appendix of Tierney [1] used unambiguous mathematical notation, the simplified equation and text included in the latter introduced some ambiguity [19]. As the majority of reported *p* values are two-sided (two-tailed), we have moved the position of the bracket in Eq. 14 to clarify that such *p* values should be halved prior to the associated *z* score being obtained [20] (see Additional file 1). If instead a one-sided (one-tailed) *p* value is reported, the *z* score may be used directly.

Importantly, the use of test statistics and *p* values in this Scenario and Scenarios 9 and 10 means that the direction of effect is not implicit. Thus, a positive or negative value must be assigned to the *O-E* according to whether the effect is in favor of the research or control arm and whether the event is favourable or not. For a favorable event (e.g., disease remission), more events and/or a shorter time to event in the research compared to the control arm suggests an effect in favor of the research intervention. For an unfavorable event (e.g., death), fewer events and/or a longer time to event on the research compared to the control arm suggests an effect in favor of the research intervention. If the *p* value is not statistically significant, or if the relative numbers of events on each arm are not reported, the separation of KM curves or textual descriptions of the results may give an indication of the direction of the results.

Using the bladder cancer example (Table 1), *V* is estimated from the number of events on each arm to be 120.87 (as per Scenario 4).

$$V= \frac{229\times 256}{485} = 120.87$$

Then, incorporating the *z* score of 1.78 (for the *p* value of 0.075) into Eq. 14 gives an *O-E* of 19.57.

$$O-E= \left(\pm \sqrt{120.87}\right)\times 1.78=\pm 19.57$$

It is clear from the trial report that survival is better in the research arm, with fewer deaths and a longer time to death. Therefore, we can assign a negative value to the *O-E* (− 19.57); and use Eq. 2 to estimate the HR as 0.85:

$${\mathrm{HR}}={\mathrm{exp}}\left[\frac{-19.57}{120.87}\right] = 0.85$$

### 9. Report presents *p* value (or chi-square statistic) and total events (randomization ratio must be 1:1)

Similarly, if just the *p* value and the total number of events are reported, and provided the ratio of participants randomized (or analyzed, if appropriate) is 1:1, then Eqs. 10 and 14 can again be used:

Based on the bladder cancer trial data, *V* is estimated to be 121.25 (as per Scenario 5) and the *O-E* is estimated to be ± 19.60:

$$\begin{array}{c}V=\frac{485}{4}=121.25\\ O-E=\left(\pm \sqrt{121.25}\right)\times 1.78=\pm 19.60\end{array}$$

Again, applying a negative value (− 19.60), then using Eq. 2 the HR is estimated to be 0.85:

$${\mathrm{HR}}={\mathrm{exp}}\left[\frac{-19.60}{121.25}\right]= 0.85$$

### 10. Report presents *p* value (or chi-square statistic), total events, and numbers randomized to each arm

Where the report presents the *p* value, the total events and the numbers randomized in each arm, Eqs. 11 and 14 can again be used, regardless of the randomization ratio:

Incorporating the bladder cancer trial data gives an estimated *V* of 121.25 (as per Scenario 6) and *O-E* of ± 19.60.

$$\begin{array}{c}V=\frac{485\times 491\times 485}{{\left(491+485\right)}^{2}} =121.25\\ O-E=\left(\pm \sqrt{121.25}\right)\times 1.78 = \pm 19.60\end{array}$$

Applying a negative sign to the *O-E,* based on the direction of the results, and using Eq. 2 provides an estimate of 0.85 for the HR:

$${\mathrm{HR}}={\mathrm{exp}}\left[\frac{-19.60}{121.25}\right] = 0.85$$

### 11. Report presents *p* value (or chi-square statistic) and CI

Although perhaps an infrequent scenario, if a *p* value (or chi-square statistic) and the associated CI for the HR are reported, it is possible to derive *V* from the CI using Eq. 7 (from Scenario 3), and the *O-E* again using Eq. 14.

In the bladder cancer example, the 95% CI of 0.71 to 1.02 gives an estimated *V* of 117.07 and an associated *O-E* of − 19.26 (after assigning a negative value, as before). Finally, a HR is calculated using Eq. 2:

$$\mathrm{HR}={\mathrm{exp}}\left[\frac{-19.26}{117.07}\right]= 0.85$$

### Further tips on extracting and using summary statistics from trial reports

#### Recognizing alternative descriptors of a HR

We have found that a reported HR (or log HR) may be described erroneously as, for example, a “risk,” “relative risk,” “incidence ratio,” or similar, particularly in older trial publications. Provided the methods section or other part of the trial report describes using a time-to-event outcome, survival analysis, time-to-event analysis, or Cox regression to generate these statistics, or if KM curves or a logrank test of treatment effect are also given, then it is almost certain that the effect measure is a HR.

If prognostic factors are being investigated alongside treatment effects, a log HR derived from the associated Cox regression may be described as a “coefficient” [1, 3]. Care is needed to identify and use the coefficient and SE that relate to the effect of the research intervention versus control (rather than any of those associated with prognostic variables).

If any doubt remains as to whether an effect measure is a HR, or that it represents the comparison of interest, it would be advisable to contact the trial investigator for clarification.

Finally, if a reported HR represents the comparison of the control versus the research arm (rather than research versus control), then reciprocals of the HR (i.e., 1/HR) and any associated CI should be used.

#### Considering varying definitions of time-to-event outcomes

Depending on the nature of the disease or condition, it may not be appropriate to combine trial results based on different outcome definitions in meta-analysis. Therefore, it is important to pay attention to such definitions in trial reports or protocols, in particular, how events are defined and how participants who have not experienced an event are censored, seeking input from trial investigators as needed. This will help to clarify whether it is reasonable to pool the trial HRs and will assist in the interpretation of the results.

#### Locating the number of events

The numbers of events needed to estimate *V* might be located in the text, in a table, or on a KM curve. Therefore, care is needed to ensure that the data used are appropriate to the desired calculation, particularly for “composite” outcomes that incorporate different types of events. For example, if a participant experiences multiple individual events and these are presented separately, there is a risk of “double counting,” or if only the first event is presented, data on subsequent events may be missing.

Only directly reported numbers of events should be used, because back-calculation these from percentages can produce rounding errors. If the number of participants who are event-free is provided, this can be used to derive the appropriate numbers of events (see “Report presents cumulative incidence or actuarial curves”).

Although rare, if the expected numbers of events for each arm are reported, these should be used preferentially to permit direct estimation of *V* [3].

- ➢ The updated version of the spreadsheet (Additional file 3) makes use of the expected numbers of events, if supplied, in a wider range of calculations than previously.

#### Choosing between adjusted and unadjusted HRs

Choosing between unadjusted or covariate-adjusted HRs is a complex issue [21] and may require consultation with an experienced statistician. To simplify matters, it is worth distinguishing between adjustment by participant characteristics and trial design features. It is common to adjust HRs for participant characteristics that are prognostic in order to maximize power [22], and incorporating similarly-adjusted HRs in meta-analysis will ensure all included trials are measuring the same effect and increase power. However, using trial HRs that have been adjusted for markedly different sets of covariates, or a mix of unadjusted and adjusted trial HRs, may increase heterogeneity and lead to a meta-analysis HR which is difficult to interpret. Therefore, in such scenarios, it may be prudent to use unadjusted trial HRs throughout.

By contrast, certain trial designs may require specific adjustments to be made, and these can differ across trials. For example, trials that used stratified block randomization or minimization should be adjusted for their stratification factors, whereas trials that used simple randomization need not be adjusted. Similarly, for a trial conducted across multiple sites, it would be appropriate to adjust for site, but for a trial carried out within a single site, such an adjustment is not possible or necessary. Thus, it is preferable to incorporate the appropriate design-specific adjusted HRs for each trial in a meta-analysis.

Note that HRs calculated from observed or expected event counts, or from KM curves are, by definition, unadjusted. Therefore, if only a covariate-adjusted HR is reported, but an unadjusted HR is required, it may be estimated using the methods described here (or, alternatively, sought directly from investigators).

#### Application of the methods to other trial designs

The methods described in this article may not be appropriate to trial designs other than parallel-group ones, at least not without additional information. For example, for a cluster-randomized trial, the methods would under-estimate the SE, because the design effect would not be accounted for [23]. In such cases, we strongly recommend obtaining estimates of a (log) HR and SE that correctly account for such design features, either from publication or directly from trial investigators.

#### Application of the methods to observational studies

The underlying methods [3] and our previous guidance [1] are geared toward the comparison of treatment arms in a randomized trial, in which allocation is controlled and confounding minimized. However, they might also be used to estimate an HR and *V* for observational studies that compare time-to-event outcomes between two exposure groups. As such groups may be imbalanced, we recommend using equations that do not require a 1:1 allocation ratio. It is worth noting that the methods cannot alleviate the risk of confounding, which is inherently greater with non-randomized studies, so covariate-adjusted or propensity score-weighted HRs are preferable in this context.

Although evidence suggests that on average there is no systematic bias in meta-analyses of observational studies [24] compared with those of randomized trials, the potential remains. Hence, we recommend assessing the impact of the study design, as well as the risk of bias [25, 26].

## Generating the HR and V from reported KM curves

The ability to indirectly estimate HRs from reported KM curves remains important, because some trial results may only be presented in this way. This relies on extracting event-free probabilities at a series of time points across such curves, estimating HRs and *V*s within each time interval, and pooling them across intervals to get an overall HR and *V* for a trial. Alongside, the minimum and maximum follow-up times or the reported numbers at risk are used to estimate the censoring pattern and hence provide appropriate denominators for the HR calculations.

While multiple steps and additional assumptions are required, if clear procedures are agreed upon and followed, then small inaccuracies or inconsistencies in data extraction or choice of intervals should have minimal impact on the estimated HR.

### 12. Report presents KM curve and follow-up information

For each time interval, it is necessary to estimate the number of participants who were (1) event-free at the start of the interval, (2) censored during the interval, (3) at risk during the interval, and (4) the number of events during the interval. These provide the means to estimate (5) the *O-E*, *V*, and HR for each time interval and then (6) estimates of the *O-E*, *V*, and HR representing the whole KM curve.

For the bladder cancer trial, the median follow-up and recruitment period (Table 1) allowed the minimum follow-up to be estimated (Additional file 2) as 14 months. Thus, censoring (step 2) is only relevant beyond this time point. Going through steps 1, 3, 4, and 5 for the previous time intervals (Table 2), the following values were estimated for the 12–15-month time interval:

**Table 2:** Data extracted from KM curve of example bladder cancer trial [17] for the outcome of survival(adapted with permission from [1])

Time at the start of interval (months)	% Event-free on research	% Event-free on control	Reported numbers at risk on research	Reported numbers at risk on control
0	100	100	491	485
6	92	92	-	-
9	86	84	-	-
12	78	75	372	355
15	73	70	-	-
18	68	63	-	-
24	62	58	283	257
33	56	52	-	-
36	54	51	200	187
48	51	46	139	132

- Event free at start of prior interval (12–15 months), research = 383.0
- Event free at start of prior interval (12–15 months), control = 363.8
- Events in prior interval (12–15 months), research = 24.6
- Events in prior interval (12–15 months), control = 24.3
- Censored in prior interval (12–15 months), research = 0.0
- Censored in prior interval (12–15 months), control = 0.0

These can be used to illustrate steps 1 to 5 for the 15–18-month interval, in the presence of censoring:

#### Step 1. Numbers event-free at start of current interval

The number of participants at the start of the current time interval is the number that were event-free at the end of the previous time interval:

15 $$\text{Event free at start of interval}=\text{Event free at start of prior interval}-\text{Events in prior interval}-\text{Censored during prior interval}$$

Therefore, data from the 12–15-month time interval are used to estimate these figures:

- Event free at start (15–18 months), research = 383.0-24.6-0=358.4
- Event free at start (15–18 months), control = 363.8-24.3-0=339.5

#### Step 2. Numbers censored during current interval

Assuming that participants are censored at a constant rate within each time interval, Eq. 16 can be used to estimate the numbers censored [1]:

16 $$\begin{array}{c} \text{Censored during interval}\\ = \text{At risk during interval}\times \frac{1}{2}\times \left(\frac{\text{End of time interval }- \text{Start of time interval}}{\text{Maximum follow-up }- \text{Start of time interval}}\right)\end{array}$$

With data from step 1, the estimated maximum follow-up of 82 months and Eq. 16, in both the research and control arms, around eight participants were estimated to be censored during the 15–18-month time interval:

$$\begin{array}{c} \mathrm{Censored}\;(15-18\;\mathrm{months}),\mathrm{research}=358.4\times\,\frac12\times\left(\frac{18-15}{82-15}\right)=8.0\\ \mathrm{Censored}\;(15-18\;\mathrm{months}),\mathrm{control}=339.5\,\times\frac12\times\left(\frac{18-15}{82-15}\right)=7.6\end{array}$$

#### Step 3. Numbers at risk during current interval, adjusted for censoring

The numbers censored can then be used to adjust (reduce) the numbers at risk during the time interval:

17 $$\text{At risk during interval, adjusted for censoring}=\text{Event free at start of interval }- \, \text{Censored during interval}$$

$$\begin{array}{c} \text{At risk during, adjusted for censoring}\;(15-18\;{\mathrm{months}}), {\mathrm{research}}=358.4-8.0=350.4\\ \text{At risk during, adjusted for censoring}\;(15-18\;{\mathrm{months}}), {\mathrm{control}}=339.5 -7.6=331.9\end{array}$$

#### Step 4. Number of events during current interval

The number of events during the interval is then estimated based on these reduced numbers at risk and the data extracted from the KM curve for that interval (Table 2):

18 $$\text{Events during interval}=\text{At risk during interval}\times\left(\frac{\%\;\text{Event free at start}-\%\;\text{Event free at end}}{\%\;\text{Event free at start}}\right)$$

$$\begin{array}{c}\text{Events during}\;(15-18\;{\mathrm{months}}), {\mathrm{research}}=350.4\times \left(\frac{73 - 68}{73}\right)=24.0\\ \text{Events during}\;(15-18\;{\mathrm{months}}), \text{control }=331.9\times \left(\frac{70 - 63}{70}\right)=33.2\end{array}$$

#### Step 5. Estimate the HR, V, and O-E for current interval

With time to event and censoring already accounted for, the formula for calculating a relative risk (risk ratio) is appropriate for estimating a HR and associated *V* within the current interval:

19 $${\mathrm{HR}}=\frac{\text{Events research}/\text{At risk research}}{\text{Events control}/\text{At risk control}}$$

20 $$V=\frac{1}{\left[\frac{1}{\text{Events research}}-\frac{1}{\text{At risk research}}+\frac{1}{\text{Events control}}-\frac{1}{\text{At risk control}}\right]}$$

The data from steps 3 and 5 and Eqs. 19, 20 and 6 give estimates of the HR, *V*, and *O-E* as 0.68, 15.17, and − 5.74, respectively:

$$\begin{array}{c}\mathrm{HR}=\frac{24.0/350.4}{33.2/331.9} =0.68\\ V=\frac{1}{\left[1/24.0-1/350.4 +1/33.2 -1/331.9\right]} =15.17\\ O-E=\mathrm{log}\left(0.68\right)\times 15.17=-5.74\end{array}$$

From these, the log HR (− 0.38) and its standard error (0.26) can also be derived, as shown in the spreadsheet.

Note that if censoring had not been accounted for, the HR for this time interval would still have been 0.68, but the *V* would have been estimated as 15.52.

#### Step 6. Combining all time intervals

The final step is to calculate the pooled HR for the trial, based on all time intervals. This involves dividing the sum of the *O-E* by the sum of the *V* values, and taking the exponential to obtain an estimated HR of 0.88.

21 $${\mathrm{HR}}={\mathrm{exp}}\left[\frac{\text{Sum of}\ \left(O-E\right)}{\text{Sum of}\ V}\right]$$

$$\begin{array}{c}\text{Sum of}\ \left(O-E\right)=0.00-5.21-3.25-0.51-5.74+\dots \mathrm{etc.}=-16.97\\ \text{Sum of}\ V=21.22+18.10+22.96+13.05+15.17+\dots \mathrm{etc.}=128.79\\ HR={\mathrm{exp}}\left[\frac{-16.97}{128.79}\right] {\mathrm{HR}}=0.88\end{array}$$

In this example, if censoring had not been accounted for, the methods would have generated the same HR, and a fairly similar *V* (136.44), probably because the trial was large and had good follow-up. By contrast, for smaller trials and/or trials with poorer follow-up, differences can be more marked.

### 13. Report presents Kaplan–Meier curve and numbers at risk

In our previous guide, we showed that the numbers at risk displayed on a KM curve offer a direct means of estimating of censoring [4]. However, this method limits data extraction to timepoints that also have accompanying numbers at risk. For example, the bladder cancer trial KM curves only display the numbers at risk annually until 5 years (Table 2).

As the numbers at risk represent the number of participants event-free at the start and end of each time interval, these quantities need not be estimated. Instead, (1) the number of participants who were at risk during the interval and (2) the number of events during the interval are needed to estimate (4) the *O-E*, *V*, and HR for each time interval. These are combined (5) to produce an *O-E*, *V*, and HR for the whole KM curve. Although not required, the number of participants who were (3) censored during the interval can be calculated for comparison with the KM curve method described in Scenario 12.

#### Step 1. Numbers at risk during the current interval

The numbers at risk and event-free probabilities at the start and end of a time interval are used to quantify the numbers of participants at risk during the interval:

22 $$\text{At risk during interval}=\frac{(\text{At risk at start}+\text{At risk at end})\times \%\;\text{Event free at start}}{\left(\%\;\text{Event free at start}+\%\;\text{Event free at end}\right)}$$

For the 0–12-month interval (Table 2):

$$\begin{array}{c}\text{At risk during}\;\mathrm{0-12}\;{\mathrm{months}}, {\mathrm{research}}=\frac{\left(491+372\right)\times 100}{100+78}=484.8\\ \text{At risk during 0-12}\;{\mathrm{months}}, {\mathrm{control}}=\frac{\left(485+355\right)\times 100}{100+75}=480.0\end{array}$$

#### Step 2. Number of events during the current interval

The same data can be used to estimate the number of events in an interval:

23 $$\text{Events in interval}= \frac{\left(\text{At risk at start}+\text{At risk at end}\right)\times \left(\%\;\text{Event free at start}-\% \;\text{Event free at end}\right)}{\% \;\text{Event free at start}+\% \;\text{Event free at end}}$$

For the 0–12-month interval, it was estimated that there were approximately 106.7 and 120.0 events in the research and control arms, respectively:

$$\begin{array}{c}\text{Events during 0-12 months, research}= \frac{\left(491+372\right)\left(100-78\right)}{\left(100+78\right)}=106.7\\ \text{Events during 0-12 months, control}=\frac{\left(355+257\right)\left(100-75\right)}{\left(100+75\right)}=120.0\end{array}$$

#### Step 3. Numbers censored during the current interval

The numbers censored are obtained from the reported numbers at risk and the event rate at the start and end of an interval:

24 $$\text{Censored during interval}=2\times \frac{ \left(\text{At risk at start} \times \%\;\text{Event free at end}\right)-\left(\text{At risk at end} \times \%\;\text{Event free at start}\right)}{\%\;\text{Event free at start }+ \%\;\text{Event free at end}}$$

Using the numbers at risk and associated event rates at 0 and 12 months, approximately 12 and 10 participants were estimated to have been censored in the research and control arms, respectively:

$$\begin{array}{c}\text{Censored during 0}-\text{12 months, research}=2\times \frac{\left(491\times 78\right)-\left(372\times 100\right)}{100+78}=12.3\\ \text{Censored during 0}-\text{12 months}, {\mathrm{control}}=2\times \frac{\left(485\times 75\right)-\left(355\times 100\right)}{100+75}=10.0\end{array}$$

If censoring had not been accounted for, the HR for this time interval would still have been 0.88, but *V* would be estimated as 65.20. Note that in Scenario 12, having estimated the minimum follow-up to be 14 months (Additional file 2), censoring that occurred in the 0–12-month time interval was missed, and so, the numbers at risk were not adjusted accordingly.

#### Step 4a. Estimate the HR and V for the current interval using the number of events and the numbers at risk during the current interval

As in Scenario 12, results from steps 1 and 2 can then be used to estimate a HR, V, and O-E for the 0–12-month time interval using Eqs. 4, 21, and 22.

#### Step 4b. Estimate the O-E and V and HR for the current interval using the numbers of events and the numbers at risk during the current interval

An alternative method estimates the expected events and then *O-E* within each interval:

25 $$\text{Expected events during, research}=\frac{\left(\text{Event research}+\text{Event control}\right)\times \text{At risk during, research}}{\text{At risk during, research}+\text{At risk during, control}}$$

Using the data for the 0–12-month interval gives an estimate for the expected events of 113.9 and *O-E* as − 7.2:

$$\begin{array}{c}\text{Expected events during, research}=\frac{\left(106.7+120.00\right)\times 484.8}{484.8+480.0}=113.9\\ O-E=106.7-113.9=-7.2\end{array}$$

Either Eqs. 9 or 10 can be used to estimate *V,* but Eq. 10 is preferred if the randomization ratio is not 1:1, or the numbers at risk during intervals are very different, e.g., if there is a large treatment effect.

$$V= \frac{226.6 \times 484.8 \times 480.0}{{\left(484.8+480.0\right)}^{2}}=56.66$$

Using Eq. 2, the HR for the interval is estimated to be 0.88.

$${\mathrm{HR}}={\mathrm{exp}}\left[\frac{-7.2}{56.66}\right]=0.88$$

#### Step 6. Combining all time intervals

Taking all time intervals and censoring into account and using Eq. 21 gives a pooled HR of 0.88 and *V* of 119.80. Thus, despite differences in the within-interval calculations, the estimates for the trial as a whole are very similar between this and the other KM curve method (Scenario 12).

### Further tips on extracting and using KM curve data from trial reports

#### Choosing the most appropriate time intervals

Previously, we advised dividing a KM curve into a series of time intervals that would give a good representation of event rates over time, with more intervals in regions of the curve where most events have occurred, and fewer intervals where fewer events have occurred [1]. However, this can lead to intervals with either no events in both arms (which means they will contribute no useful data) or no events in one arm. Both cause a division-by-zero problem in estimating *V* for an interval. While a small correction factor was incorporated in the original spreadsheet to account for this, zero events can lead to greater estimation error and are best be avoided, particularly for small trials.

For the ovarian cancer trial, our prior HR estimate was 1.21 (95% CI 0.62–2.36) [1], but by selecting fewer intervals and thereby avoiding zero events, we obtained a HR of 1.52 (95% CI 0.0.90–2.56), much closer to that calculated directly (HR = 1.51, 95% CI 0.90–2.53). Thus, if intervals with zero events arise, we recommend collapsing adjacent intervals to resolve the issue.

Having extracted data from a large number of KM curves [27], it became clear that where events happen quickly, such as in advanced cancer, allowing up to a 20% event rate within an interval (as suggested previously [1, 3]) can lead to oversimplification of the pattern of events. Hence, we now recommend that the event rate within an interval should be no more than 15% and no less than 5%. When it is not possible to follow this advice, for example, due to a large treatment effect, we suggest accepting a higher event rate in one arm to avoid the potentially greater issue of zero events in the other. Seemingly, this may lead to too few intervals, but for small trials, or trials with few events, this is usually appropriate. That said, if only two or three clinically relevant time points are provided in a trial report, they are unlikely to represent the entire KM curve adequately, and so should not be used to estimate a HR.

A number of “graph digitizer” software packages allow data to be extracted more easily and accurately from digital images of published KM curves than if done manually [28–30]. These have been designed primarily to allow large numbers of data points to be extracted automatically for the purpose of reconstructing a KM curve. If they are used, instead, to assist in estimating a HR and *V*, we still recommend carefully selecting that a limited number of these to ensure robust estimation.

Table 3 summarizes our advice for best practice.

**Table 3:** Best practice for data extraction from reported Kaplan–Meier curves

• Agree KM curve method in advance of data extraction
- Choose to use the KM curve and information on follow-up (Scenario 12)
- Choose to use the KM curve and numbers at risk (Scenario 13)
- Choose to use both KM methods (e.g., for comparison)
- But allow flexibility to deviate if specific issues arise (e.g., zero events in an arm or too few intervals with numbers at risk)
• For Scenario 12, agree on the time intervals in advance of data extraction
- Select intervals guided by the shape of the KM curve (i.e., the event rates), rather than at regular time points
- Select more time intervals where more events have occurred (i.e., the curves are steeper). Aim to include not more than a 15% event rate in an interval
- Select fewer intervals where fewer events have occurred (i.e., the curves are flatter). Aim to include at least a 5% event rate in an interval
- Avoid time intervals with zero events in one or both arms—make the time interval larger to avoid this
• For Scenario 13, select only time intervals that have accompanying numbers at risk
• For both scenarios, avoid extracting data at time points where few or no events have occurred (i.e., the curves have leveled out)
• Duplicate or cross-check data extracted by another researcher

#### Dealing with cumulative incidence or actuarial curves

If the cumulative incidence of an event (e.g., [31]) on research versus control is displayed on a curve, data can be extracted in a similar fashion to that from traditional KM curves. However, these data will represent event probabilities rather than event-free probabilities, so will need to be subtracted from 1 before being entered into the spreadsheet. Note this will generate a trial HR for the time to the event (rather than the cumulative incidence).

As the KM curve method assumes that censoring occurs at a constant rate between the minimum and maximum follow-up times, it is consistent with the actuarial life-table method (as used, for example, in [32]), in which withdrawals are assumed to occur uniformly within each interval. Although the life-table method also assumes that events occur uniformly, whereas the KM curve provides information on the exact timings of events, our view is that the KM curve method will give sensible results, but formal evaluation would need to confirm this.

#### Consideration of non-proportional hazards

Standard time-to-event analysis methods that generate HRs, such as Cox regression, assume that the ratio of hazards is constant over time (“proportional hazards”). However, this assumption may not be correct: for example, if effects attenuate once a treatment is completed, or if participants cross over from the control to the research treatment at disease progression. Sometimes non-proportionality of hazards will be apparent from visual inspection of published KM curves, for example, if they cross or are “banana-shaped.” Alternatively, statistical methods are available for exploring the non-proportionality of hazards, such as testing for an interaction between the estimate of the (log) HR and time [33] or the Grambsch-Therneau family of tests on the Schoenfeld residuals [34], but these require either individual participant data (IPD) or “pseudo” IPD generated using KM curve reconstruction methods [35–37].

If non-proportional hazards arise because of a quantitative change in the size of an effect over time, a HR may still be a reasonable summary statistic (e.g., of an “average” treatment effect over time). This is because a HR may be viewed as the average of (censoring-adjusted) risk ratios calculated at each event time [3], and typically, the risk of bias appears unaffected by the use of a HR in this context [37]. However, in the case of more substantial departures from proportionality, such as a qualitative change in the direction of an effect over time (i.e., the HR changes from less than 1 to greater than 1 or vice versa), a trial HR may become uninterpretable.

In the context of meta-analysis, heterogeneity of trial HRs may be increased with even minor departures from proportionality, if trials have markedly different accrual or follow-up durations. Substantial non-proportionality of hazards in one or more trials may render a meta-analysis HR inappropriate or difficult to interpret. In such instances, the use of alternative summary statistics, such as the restricted mean survival time (RMST) [38], may be preferable, but will likely require KM curve reconstruction [35–37].

The methods described here are designed specifically to estimate HRs under standard assumptions and typical scenarios. While they are not applicable in the context of competing risks, indirect estimation of Fine-Gray [39] sub-distribution HRs (or similar) may be possible given sufficient information.

#### Estimating absolute effects from a hazard ratio

As the HR is a relative effect measure, it may translate to different absolute effect sizes, depending on the baseline (control-arm) event rate. Therefore, it can be useful to examine absolute differences in the proportion of participants who are event-free at one or more clinically relevant time points, or for groups of participants with different underlying prognoses (i.e., have different control arm event rates). Assuming proportional hazards:

26 $$\text{Difference in event-free probability}=\mathrm{exp}\left[\mathrm{log}\left(\text{proportion event-free}\right)\times {\mathrm{HR}}\right]-\left(\text{proportion event-free}\right)$$

Using data from the bladder cancer example (Table 2), the estimated percentage of participants surviving (i.e., event-free) in the control arm at 2 years was 58%. Using this and the HR of 0.85 in Eq. 26 gives a 5% (0.05) absolute improvement in survival at 2 years. In other words, survival was increased from 58% with the control treatment to 63% with the research treatment:

$$\text{Difference in event-free probability}=\mathrm{exp}\left[\mathrm{ln}\left(0.58\right)\times 0.85\right]-0.58=0.05$$

Alternatively, assuming an exponential distribution for the event times, a HR can be translated into an absolute difference in the median time event-free between arms. Given an estimated median time for the control arm, Eq. 27 can be used to obtain an estimated median time event-free for the research arm. Then, Eq. 28 allows estimation of the difference in medians between the research and control arms:

27 $$\text{Median time event-free, research}=\frac{\text{Median time event-free, control}}{\textrm{HR}}$$

28 $$\text{Difference in median time event-free}=\text{Median time event free, research}-\text{Median time event free, control}$$

The reported median survival in the control arm for the bladder cancer trial was estimated to be 37.5 months, which gives a median survival in the research arm of 44.1 months. Hence, the absolute improvement in median survival with the research treatment is 6.6 months:

$$\begin{aligned}\text{Median time event-free, research}&=\frac{37.5}{0.85}=44.1\\ \text{Difference in median time event-free}&=44.1-37.5=6.6\end{aligned}$$

These approaches require an initial estimate for the control arm, of either the proportion of participants event-free or of the median time event-free. Such information might be obtained from a KM curve in a representative trial or meta-analysis, from epidemiological data, or from other sources. Furthermore, these approaches assume that event-free survival times follow an exponential distribution, which is often reasonable, but not guaranteed. Note that we do not recommend using median survival times to estimate a HR, as this has been shown to be inaccurate [40, 41].

## Discussion

We have provided updated practical guidance on estimating HRs and related statistics from summary time-to-event data presented in trial reports, including additional scenarios that researchers might face, clarification of ambiguities, and further advice on data extraction. This is complemented by an updated and enhanced calculations spreadsheet that generates all the summary statistics required for pooling HRs in meta-analysis.

### Strengths

This guide builds on our extensive experience of using these methods in practice, which has increased our understanding substantially and allowed us to refine our approaches and the spreadsheet accordingly. Moreover, we have taken account of practical issues faced by researchers when estimating HRs and related statistics from published summary data. While the CONSORT statement [42] has likely improved reporting of trial time-to-event analyses, they can still be presented in multiple ways [43], so this improved guidance and spreadsheet now incorporates a wider range of scenarios. Supplemental spreadsheets and code offering alternative methods for estimating HRs are available on request.

### Limitations

This guidance cannot help researchers rectify issues with the analysis of individual trials, inadequate descriptions of analytical methods, limited or variable follow-up, unclear or crossing KM curves, or biases associated with selective reporting of trial results. In such instances, or where the published data are otherwise insufficient, we recommend seeking further information and/or appropriate data direct from trial investigators, for all outcomes and participant subgroups of interest [44]. Doing so has permitted a more thorough and reliable meta-analysis of time-to-event outcomes in prostate cancer [45–48] and COVID-19 [49]. Bear in mind, however, that the collection of individual participant data offers the best opportunity to access updated follow-up and conduct detailed analyses of the effects of treatments on time-to-event outcomes, including testing of assumptions, adjusting for covariates, and in-depth analysis of effect modifiers [50–52].

### Context

The Parmar methods [3] for estimating HRs from KM curves, upon which this and the previous guidance [1] is based, estimates HRs independently and combines them via standard “inverse-variance,” fixed-effect meta-analysis [18] to provide a single HR representing the whole trial. Others have proposed an alternative approach [43] using Mantel–Haenszel methods [18] to combine HRs across time intervals. Although simulation results suggest that this approach is better than the Parmar methods for small trials with low event rates [53], this may have been due to the presence of intervals with zero events, which we now strongly recommend avoiding. Another proposed method [54] aims to better approximate the true censoring distribution when numbers at risk are available only for a limited number of timepoints. Data are extracted from KM curves both at time points where numbers at risk are presented, and also at selected time points in between, and a parametric interval-censoring model is used to account for the lack of information between timepoints.

Graph digitization software [28–30] and “data reconstruction” methods [35, 54, 55] have increased the ability to analyze the content of reported KM curves, but their usefulness for the estimation of HRs has received less attention. One approach based on the product-limit estimator [35] may outperform the Parmar methods, but requires data from every “step” of a KM curve (which may be unworkable for larger studies or poor-quality images) as well as the numbers at risk for at least two timepoints [3, 35]. In a recent comparison between approaches [53], the Parmar methods showed satisfactory levels of accuracy, without needing to rely on accurate digitization of high-resolution images. The authors did note that a lack of clear guidance on selecting time intervals for the Parmar methods may have led to variation in results, which we try to rectify in this article. Furthermore, there is evidence both from empirical studies [4, 27, 56] and from simulation studies [43, 53] that there is no systematic bias in HRs estimated from the KM curve method in comparison to direct or other indirect methods [27]. That said, image quality, the subjective choice of time intervals, and the assumptions and estimations made when handling follow-up and censoring will continue to be sources of variation and potential inaccuracy for any method that relies on the extraction of data from KM curves.

## Conclusions

This update to our previous guidance and accompanying spreadsheet will provide valuable additional tools for those producing meta-analyses of published, summary time-to-event data. With methods continually evolving, we will continue to log queries and explore ways to keep our advice and software up-to-date, informative, and practical.

## Supplementary Information

Additional file 1. Mathematical formulae for estimating HR and V from data.

Additional file 2. Mathematical formulae for estimating HR and V from KM curves.

Additional file 3. Calculations spreadsheet version 7.0.

Additional file 4. Spreadsheet guide.

## Data Availability

Data sharing is not applicable to this article as no datasets were generated or analyzed during the current study.
