# Correction: Practical methods for incorporating summary time-to-event data into meta-analysis: updated guidance

**DOI:** 10.1186/s13643-026-03241-7

**Published:** 2026-07-11

**Authors:** Jayne F. Tierney, Sarah Burdett, David J. Fisher

**Affiliations:** MRC Clinical Trials Unit, Medical Research Council Clinical Trials Unit, University College London, London, UK

**Correction: Syst Rev 14, 84 (2025)**

**https://doi.org/10.1186/s13643-025-02752-z**

Following publication of the original article [1]. The authors found that editorial notes were unfortunately captured in the body of the article, specifically under the second paragraph of the Context section.

The error is highlighted below in bold. The original article [1] has been corrected.

**Current sentence read as:** The authors did note that a lack of clear guidance on selecting time intervals for the Parm**published, JT and DF have answered many queriespublished, JT and DF have answered many queries**ar methods may have led to variation in results, which we try to rectify in this article.

**Correct sentence should read as:** The authors did note that a lack of clear guidance on selecting time intervals for the Parmar methods may have led to variation in results, which we try to rectify in this article.
