# Supplementary material for: Practical methods for incorporating summary time-to-event data into meta-analysis: updated guidance
Source: Syst Rev. 2025 Apr 10;14:84. doi: 10.1186/s13643-025-02752-z (PMC11984287; doi:10.1186/s13643-025-02752-z)
Supplement: Supplementary file 1 — Additional file 1. Mathematical formulae for estimating HR and V from data. [file 13643_2025_2752_MOESM1_ESM.docx]

# **Summary and derivation of mathematical formulae for estimating HR and V**

## **Introduction**

This section summarizes information given in the main article (Tierney et al. *Systematic Reviews* 2024). In the main article, formulae were described and justified in a manner designed to be accessible to a wide audience, and in places expressions were given using descriptive phrases. By contrast, all formulae in the present document are given using formal mathematical notation.

As in the main article, formulae are presented consistently in terms of HR, *O-E* and (logrank) *V*, with changes and additions documented. Some formulae in our previous article (Tierney et al. *Trials* 2007) were presented in terms of log HR and *V** (that is, the log hazard ratio and its variance), where *V* and *V** are reciprocals of each other. To avoid confusion between *V* and *V**, here we do not use the latter, instead rearranging in terms of V or, occasionally, the standard error of the log HR (abbreviated as StdErr). Index *i* denotes a quantity relating to a specific trial (in contrast to an overall value pooled across trials), whilst indices *r* and *c* denote research and control arms respectively.

Throughout this section, where a formula was given previously (albeit with updated descriptions in some instances), the same equation number is provided with prefix “AP”. New equations included are clearly described as such. This Additional File describes direct and indirect methods using equations involving reported quantities (Scenarios 1 to 11). Additional File 2 describes methods for estimating HR and *V* algorithmically from Kaplan-Meier (KM) curves (Scenarios 12 and 13).

## **Scenarios 1 and 2: Direct estimation**

### Scenario 1: Direct estimation of HR and V using expected event counts

Formally, HR and *V* may be defined using expected event counts, as follows (where Equation AP6 follows from taking the log of the expression in Equation AP5, and then applying standard algebra for variances of random variables):

| Equivalent of equation AP5: | $\mathrm{HR}_{i}=\frac{{O_{ri}}/{E_{ri}}}{{O_{ci}}/{E_{c}}}$ |
| --- | --- |
| Equivalent of equation AP6: | $\text{V}_{i}=\frac{E_{ri}E_{ci}}{E_{ri}+E_{ci}}$ |

From here, estimation of *O-E* follows straightforwardly:

| New equation: | $O_{ri}-E_{ri}=ln(\mathrm{HR}_{i})\times\text{V}_{i}$ |
| --- | --- |

### Scenario 2: Direct estimation of HR, O-E and V using the logrank method

As previously, the HR may instead be estimated using the logrank method, which makes direct use of *V*. Given *O* and *E*, we derive the HR as follows:

| Equivalent of equation AP7: | $\text{HR}_{i}=\text{exp}\left[ \frac{O_{ri}-E_{ri}}{V_{i}} \right]=\text{exp}\left[ \frac{O_{ci}-E_{ci}}{V_{i}} \right]$ |
| --- | --- |
| Equivalent of equation AP7: | $V_{i}=\frac{O_{ri}-E_{ri}}{\log{HR}_{i}}=\frac{O_{ci}-E_{ci}}{\log{HR}_{i}}$ |

If not reported, *V* may be estimated from the standard error of the log HR (note that this is simply a re-statement of the relationship between *V* and *V** given in the Introduction):

| New equation: | $V_{i}=\frac{1}{{\text{se}\left[ ln(\mathrm{HR}_{i}) \right]}^{2}}$ |
| --- | --- |

### Scenarios 3 to 7: Indirect estimation of V (given HR or O-E)

In this section, we assume that *O-E* or HR are available, either as reported or available directly via Scenarios 1 or 2 above. We assume that V is not available directly, and therefore needs to be estimated from other available information.

### Scenario 3: Use of a confidence interval

As previously, indirect estimation of *V* from a confidence interval, where *α* is the significance level of the confidence interval for the log HR. The most common value for *α* is 0.05 (that is, 5% significance), in which case the value returned by the standard Normal inverse cumulative distribution function $\Phi^{-1}$ is approximately 1.96. In the expression below, UpperLogCI and LowerLogCI denote the reported upper and lower confidence limits for the log HR. Hence, if a confidence interval for the (exponentiated) HR is reported, the logarithm of those values would need to be taken before entering them into the equation.

| Equation AP10: | $V_{i}=\left[ \frac{2\times\Phi^{-1}\left( 1-\frac{\alpha_{i}}{2} \right)}{\text{UpperLogCI}_{i}-\text{LowerLogCI}_{\text{i}}} \right]^{2}$ |
| --- | --- |

### Scenario 4: Use of number of events in each arm

Indirect estimation of *V* from the number of events in each arm, where $O_{i}$ denotes the total of the events observed in the research and control arms for a specific trial. Note that this does *not* require the randomisation ratio to be 1:1, in contrast to the statement made in ([1](#_ENREF_1)). Equation AP11 is derived from Equation AP6 by considering that, under the null hypothesis, observed and expected numbers of events should be roughly equal; we therefore simply replace one with the other.

| Equation AP11: | $V_{i}={O_{ri}O_{ci}}/{O_{i}}$ |
| --- | --- |

### Scenario 5: Use of total number of events (assumes 1:1 allocation)

As previously, indirect estimation of *V* from the total number of events. To re-iterate, the randomisation ratio must be 1:1. In that case, then under the null hypothesis we might expect the number of observed events to be roughly equal in treatment and control arms. Setting those quantities to be exactly equal in Equation AP11, we arrive at the approximation for V given by Equation AP12:

| Equation AP12: | $V_{i}={{O_{i}}}/4$ |
| --- | --- |

### Scenario 6: Use of total events and numbers analysed in each arm

Here, we proceed along similar lines to Scenario 5 but without assuming a 1:1 randomisation ratio. Under the null hypothesis, we might expect the number of observed events to be scaled in proportion to patients analysed. That is, the ratio of observed events to patients in the control arm is equal to that ratio in the research arm. Applying this assumption to Equation AP11, we arrive (after some algebra) at Equation AP13, which requires just the total number of events across both arms, together with the individual numbers of participants analysed in each arm, denoted by $R_{ri}$ and $R_{ci}$.

| Equation AP13: | $V_{i}=\frac{O_{i}R_{ri}R_{ci}}{\left( R_{ri}+R_{ci} \right)^{2}}$ |
| --- | --- |

### Scenario 7: Use of HR and p-value

This is a new addition. The notes below, regarding use of the associated test statistic in place of the p-value, are also relevant here. This equation may be derived by combining Equation AP7 with the expression on the following page demonstrating the relationship between the logrank test chi-squared statistic and its p-value.

| New equation | $V_{i}={\left[ \Phi^{-1}\left( 1-\frac{p_{i}}{2} \right) \right]^{2}}/{\left( {\log HR}_{i} \right)^{2}}$ |
| --- | --- |

### Scenarios 8 to 11: Indirect estimation of O-E

In this section, we assume that neither O-E nor HR are available directly. Therefore, assuming that V is also not available directly, we proceed in two steps:

1. Estimation of *O-E* as a function of (as yet unknown) *V*
2. Estimation of *V* as for Sections 3 to 7.

Finally, we may then estimate HR using Scenario 2. Note that the use of test statistics and p-values means that the direction of effect is not implicit to the calculations, and must be supplied explicitly by the user. If the effect appears to be in favour of the research arm, then *O-E* should be a negative value. If the effect is favour of control, then *O-E* should be a positive value.

If the associated test statistic (typically a log-rank chi-squared statistic on 1 degree of freedom) is given alongside the p-value, it will typically be a larger number and may be reported to a greater degree of accuracy. Therefore, alternative expressions may instead be used, based on the following relationship between the logrank test chi-squared statistic and its p-value:

| New equation | $\chi_{\text{logrank}}^{2}=\frac{\left( O_{ri}-E_{ri} \right)^{2}}{V_{i}}=\left[ \Phi^{-1}\left( 1-\frac{p_{i}}{2} \right) \right]^{2}$ |
| --- | --- |

where $\Phi^{-1}$ represents the inverse cumulative distribution function of the standard Normal distribution (see also Equation AP10 above).

**Step (a): Indirect estimation using a p-value or chi-square statistic**

| Part of Equations AP14, AP15, AP16: | $O_{ri}-E_{ri}=\left[ \text{direction of effect} \right]\times\Phi^{-1}\left( 1-\frac{p_{i}}{2} \right)\times\sqrt{V_{i}}$ |
| --- | --- |

**Step (b): Estimation of *V* as for Sections 3 to 7**

In the following formulae, we simply substitute the relevant formula for *V* into the expression given for Step (a). Note that, in the main article, Steps (a) and (b) and associated equations are kept separate to in order to simplify the individual equations. However, the expressions given here are exactly equivalent.

Using total observed events, assuming 1:1 allocation ratio (Scenario 8):

| Equation AP15: | $O_{ri}-E_{ri}=\left[ \text{direction of effect} \right]\times\Phi^{-1}\left( 1-\frac{p_{i}}{2} \right)\times\frac{1}{2}\sqrt{O_{i}}$ |
| --- | --- |

Using observed events in each arm (Scenario 9):

| Equation AP14: | $O_{ri}-E_{ri}=\left[ \text{direction of effect} \right]\times\Phi^{-1}\left( 1-\frac{p_{i}}{2} \right)\times\sqrt{\frac{O_{ri}O_{ci}}{O_{i}}}$ |
| --- | --- |

Using numbers analysed in each arm (Scenario 10):

| Equation AP16: | $O_{ri}-E_{ri}=\left[ \text{direction of effect} \right]\times\Phi^{-1}\left( 1-\frac{p_{i}}{2} \right)\times\frac{\sqrt{{O_{i}R}_{ri}R_{ci}}}{\left( R_{ri}+ R_{ci} \right)}$ |
| --- | --- |
