# Supplementary material for: Practical methods for incorporating summary time-to-event data into meta-analysis: updated guidance
Source: Syst Rev. 2025 Apr 10;14:84. doi: 10.1186/s13643-025-02752-z (PMC11984287; doi:10.1186/s13643-025-02752-z)
Supplement: Supplementary file 2 — Additional file 2. Mathematical formulae for estimating HR and V from KM curves. [file 13643_2025_2752_MOESM2_ESM.docx]

# **Methods for estimating HR and V from Kaplan-Meier curves**

## **Introduction**

This section re-summarizes information given in the main article, where formulae were described and justified in a manner designed to be accessible to a wide audience, and in places expressions were given using descriptive phrases. By contrast, all formulae in the present document are given using formal mathematical notation.

This Additional File describes methods for estimating HR and V algorithmically from Kaplan-Meier (KM) curves (Scenarios 12 and 13). Additional File 1 describes direct and indirect methods for estimating HR and V using equations involving reported quantities (Scenarios 1 to 11).

## **Scenarios 12 and 13**

### **Scenario 12: Numbers at risk not available**

As described previously (Tierney et al. *Trials* 2007), for a KM curve summarizing a specific trial *i* we begin by considering *T* distinct time points ($\tau=1,\ldots,T$) obtained following the advice given under “Choosing the most appropriate time intervals”, which define *T* – 1 non-overlapping time intervals:

| $t=\text{whole time interval }\left( \tau-1,\tau\right)$ |
| --- |
| $t_{s}=\text{start of the time interval }\left( \tau-1,\tau\right)$ |
| $t_{e}=\text{end of the time interval }\left( \tau-1,\tau\right)$ |

We next define the effective numbers of patients at risk, events and censorings during each time interval as follows:

| Effective number of patients at risk during time interval $\left( \tau-1,\tau\right)$ | Research arm: $R_{ri}\left( t \right)$  Control arm: $R_{ci}\left( t \right)$ |
| --- | --- |
| Effective number of events during time interval $\left( \tau-1,\tau\right)$ | Research arm: $D_{ri}\left( t \right)$  Control arm: $D_{ci}\left( t \right)$ |
| Effective number of patients censored during time interval $\left( \tau-1,\tau\right)$ | Research arm: $C_{ri}\left( t \right)$  Control arm: $C_{ci}\left( t \right)$ |
| Event-free probability at the start of time interval $\left( \tau-1,\tau\right)$ | Research arm: $S_{ri}\left( t_{s} \right)$ |
| Event-free probability at the end of time interval $\left( \tau-1,\tau\right)$ | Research arm: $S_{ri}\left( t_{e} \right)$ |

To begin, we set the values of $R_{ri}\left( t_{s} \right)$ and $R_{ci}\left( t_{s} \right)$ to be equal to the total observed numbers of patients analysed within each arm. We then proceed iteratively, within each arm separately. The formulae below are written in terms of the research arm, but equivalent calculations also need to be performed for the control arm. For each time interval, our first task is to estimate the effect number of patients censored during the interval. The formula below is derived from assuming a constant censoring rate within the interval, so that a triangle is formed; see (2):

$$C_{ri}\left( t \right)=R_{ri}\left( t_{s} \right)\times\frac{1}{2}\times\frac{t_{e}-t_{s}}{F_{\text{max}}-t_{s}}$$

…where $F_{\text{max}}$ denotes the maximum follow-up time. This allows us to estimate the effective number at risk during the time interval, adjusted for censoring:

$$R_{ri}\left( t \right)=R_{ri}\left( t_{s} \right)-C_{ri}\left( t \right)$$

With reference to the event-free proportions read off from the KM curve at the start and end of the current time interval, we next estimate the effective number of events during the interval:

$$D_{ri}\left( t \right)=R_{ri}\left( t \right)\times\left( \frac{S_{ri}\left( t_{s} \right)-S_{ri}\left( t_{e} \right)}{S_{ri}\left( t_{s} \right)} \right)$$

Finally, we may estimate the effective number event-free at the start of the *next* time interval:

$$R_{ri}\left( t_{s} \right)=R_{ri}\left( t-1 \right)-D_{ri}\left( t-1 \right)-C_{ri}\left( t-1 \right)$$

We continue in this fashion across all time intervals for which $t_{s}\geq F_{\text{max}}$ and $F_{\text{min}}\leq t_{e}\leq F_{\text{max}}$, where $F_{\text{min}}$ and $F_{\text{max}}$ denote the minimum and maximum follow-up time, respectively. Having done so for both arms, we are able to estimate the HR and *V* within each time period:

$${HR}_{i}\left( t \right)=\frac{{D_{ri}\left( t \right)}/{R_{ri}\left( t \right)}}{{D_{ci}\left( t \right)}/{R_{ci}\left( t \right)}}, V_{i}\left( t \right)=\frac{1}{\left[ \frac{1}{D_{ri}\left( t \right)}-\frac{1}{R_{ri}\left( t \right)}+\frac{1}{D_{ci}\left( t \right)}-\frac{1}{R_{ci}\left( t \right)} \right]}$$

Because we have accounted for censoring within each interval, each ${HR}_{i}\left( t \right)$ applies to a fixed risk set, and we may calculate an overall HR and V by pooling across intervals using standard methods for fixed-effects inverse-variance meta-analysis.

### **Scenario 13: Numbers at risk available**

If numbers at risk are available, we need to introduce some different notation. Let $j=1$ denote the control arm, and $j=2$ denote the research arm. Let $t_{i}$ and $t_{i-1}$ denote the time at the start of the current and previous interval respectively. We then define the following quantities:

| Known: |  |
| --- | --- |
| Number at risk at end of interval $\left[ t_{i-1}, \right.\left. t_{i} \right)$ in arm *j* | $n_{j,i}$ |
| Number at risk at start of interval $\left[ t_{i-1}, \right.\left. t_{i} \right)$ in arm *j* | $n_{j,i-1}$ |
| Event-free probability at end of interval $\left[ t_{i-1}, \right.\left. t_{i} \right)$ in arm *j* | $s_{j,i}^{*}$ |
| Event-free probability at start of interval $\left[ t_{i-1}, \right.\left. t_{i} \right)$ in arm *j* | $s_{j,i-1}^{*}$ |
| Unknown: |  |
| Number at risk during interval $\left[ t_{i-1}, \right.\left. t_{i} \right)$ in arm *j* | $n_{j,i}^{*}$ |
| Number at events during interval $\left[ t_{i-1}, \right.\left. t_{i} \right)$ in arm *j* | $d_{j,i}^{*}$ |
| Number censored during interval $\left[ t_{i-1}, \right.\left. t_{i} \right)$ in arm *j* | $c_{j,i}^{*}$ |
| Logrank expected events during interval $\left[ t_{i-1}, \right.\left. t_{i} \right)$ in arm $j=2$ (research arm) | $e_{j,i}^{*}$ |

Unlike the method where numbers at risk are not available, here we do not need to proceed iteratively, but can instead estimate HR and V independently within each time interval. In the list above, the unknown quantities may be estimated from the known quantities by assuming a constant event rate and censoring rate within each interval, as follows:

$$n_{j,i}^{*}=\frac{\left( n_{j,i-1}+n_{j,i} \right)}{\left( s_{j,i-1}^{*}+s_{j,i}^{*} \right)}s_{j,i-1}^{*}$$

$$d_{j,i}^{*}=\frac{\left( n_{j,i-1}+n_{j,i} \right)}{\left( s_{j,i-1}^{*}+s_{j,i}^{*} \right)}\left( s_{j,i-1}^{*}-s_{j,i}^{*} \right)$$

$$c_{j,i}^{*}=2\frac{\left( n_{j,i-1}s_{j,i}^{*}-n_{j,i}s_{j,i-1}^{*} \right)}{\left( s_{j,i-1}^{*}+s_{j,i}^{*} \right)}$$

$$e_{2,i}^{*}=\left( d_{j,i-1}^{*}+d_{j,i}^{*} \right)\frac{n_{2,i}^{*}}{n_{2,i}^{*}+n_{1,i}^{*}}$$

We may now equate $e_{2,i}^{*}$ with $D_{ri}\left( t \right)$ and $n_{j,i}^{*}$ with $R_{ri}\left( t \right)$, and proceed to estimate HR and V as in the method where numbers at risk are not available.

## **Estimating or ‘guesstimating’ minimum and maximum follow-up times**

As described previously (Tierney et al. *Trials* 2007), if not explicitly reported the minimum and maximum follow-up for a trial may be estimated from other reported indicators of the level of follow-up. These may include the accrual period (the time between the first and last participant being entered into the trial, the median follow-up or the date of submission of the trial manuscript to a journal (often found on the first or last page of a trial publication).

The following are some strategies that may be used for each, in descending order of preference:

### **Estimating minimum follow-up**

If the trial report presents:

1. Censoring tick marks on Kaplan-Meier curve

- Assume first tick mark indicates the point of minimum follow-up

1. Median follow-up and accrual period

- Assume minimum follow-up = median follow-up minus half the accrual period

1. Date of analysis and accrual period

- Assume minimum follow-up = date of analysis minus final date of accrual

1. Date of submission and accrual period

- Assume estimated date of analysis = date of submission minus 6 months
- Assume minimum follow-up = estimated date of analysis minus final date of accrual

### **Estimating maximum follow-up**

If the trial report presents:

1. Censoring tick marks on Kaplan-Meier curve

- Assume last tick mark indicates the point of maximum follow-up, provided it is clearly stated that curve has been drawn to the end of follow-up, as it may have been drawn only as far as is reliable. If the curves have levelled out, it is likely that events have stopped happening and/or the numbers at risk are very small.

1. Median follow-up and accrual period

- Assume maximum follow-up = median follow-up plus half the accrual period

1. Date of analysis and accrual period

- Assume maximum follow-up = date of analysis minus first date of accrual

1. Date of submission and accrual period

- Assume estimated date of analysis = date of submission minus 6 months
- Assume maximum follow-up = estimated date of analysis minus first date of accrual

Whichever process is used for estimating minimum and maximum follow-up times, we recommend that this clearly recorded in the spreadsheet for future reference.

The updated Spreadsheet provides a “Comments” box on the “Trial Details” sheet that can be used to log details of how the minimum and maximum follow-up were estimated.
