# Supplementary material for: Practical methods for incorporating summary time-to-event data into meta-analysis: updated guidance
Source: Syst Rev. 2025 Apr 10;14:84. doi: 10.1186/s13643-025-02752-z (PMC11984287; doi:10.1186/s13643-025-02752-z)

## **Guide** **to** **using** **the** **calculations** **spreadsheet**

### Navigating the spreadsheet tabs

Accompanying this article, we provide an updated spreadsheet that can be downloaded and used to perform all the calculations described (see Additional File 3). Furthermore, it allows users to log all the relevant information pertaining to estimation of a trial HR in one place. To ensure familiarity and continuity for users of the previous version ([1](#_ENREF_1)), we have kept changes to a minimum.

The *Cover sheet* (Figure AF3.1a) provides references for users to cite when they use the spreadsheet, the contents, a summary of how it works and a list of those who contributed to spreadsheet development.

In the *Trial details sheet* (Figure AF3.1b) users can enter descriptions of the trial or study (which then populates subsequent sheets), details of the researcher entering the information, the source(s) of the extracted data and any additional comments. Some general instructions for entering data on this and subsequent sheets are provided.

The *Summary data sheet* (Figure AF3.2) allows users to enter all the available information and summary statistics.

In the *Curve data sheet*, the number of participants randomised or analysed (i.e. event-free at the start), the selected time points and the associated event-free probabilities for the research and control arms should be entered, together with the actual or estimated minimum and maximum follow-up (Figure AF3.3a). The time intervals will be generated automatically from the timepoints, and the inputted data will be displayed on the accompanying curve copy sheet (Figure AF3.3b). Alternatively, similar data from a KM and the available numbers at risk can be input in the *Curve data with n(risk) sheet*.

Numbers should be entered into the spreadsheet in accordance with country-specific Excel settings. For example, in the UK and United States, a decimal point is represented by a full stop or period (e.g., “24.55”), whereas in many others it is represented by a comma (e.g. “24,55”).

Once all the summary statistics and/or curve data are entered, all possible estimates of the HR, CI, log HR, standard error of log HR, *V* and *O-E* will be displayed in the *Output Information sheet* (Figure 4), with other cells left blank. The included key clarifies which data and methods are used for each set of estimates. To accommodate additional scenarios, 13 methods are included rather than the 11 described previously ([1](#_ENREF_1)), and these are slightly re-ordered as per Additional File 1.

## Figure AF3.1: Calculations spreadsheet: (a) Cover sheet, (b) Trial details sheet

(a)


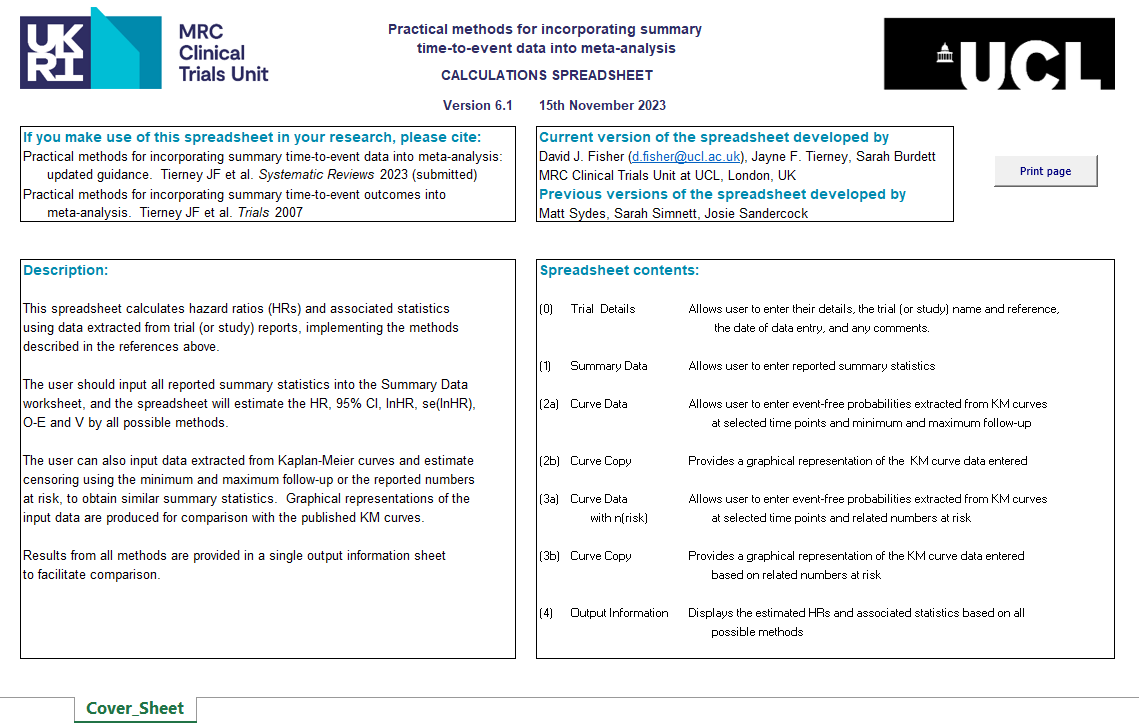


(b)


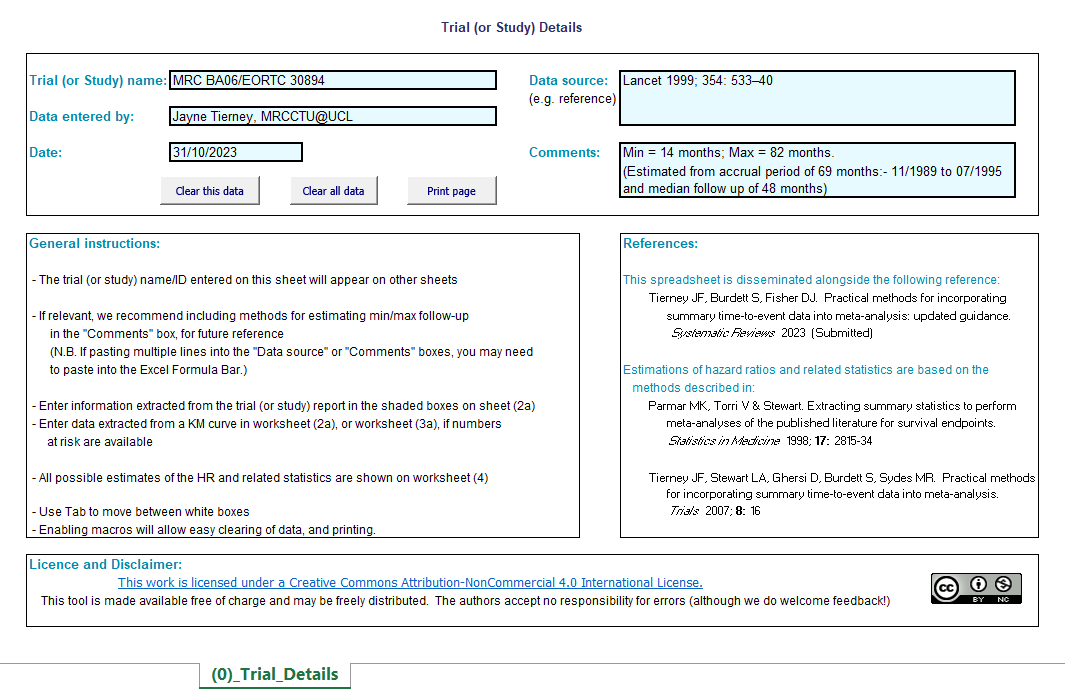


## Figure AF3.2: Calculations spreadsheet: Summary data sheet


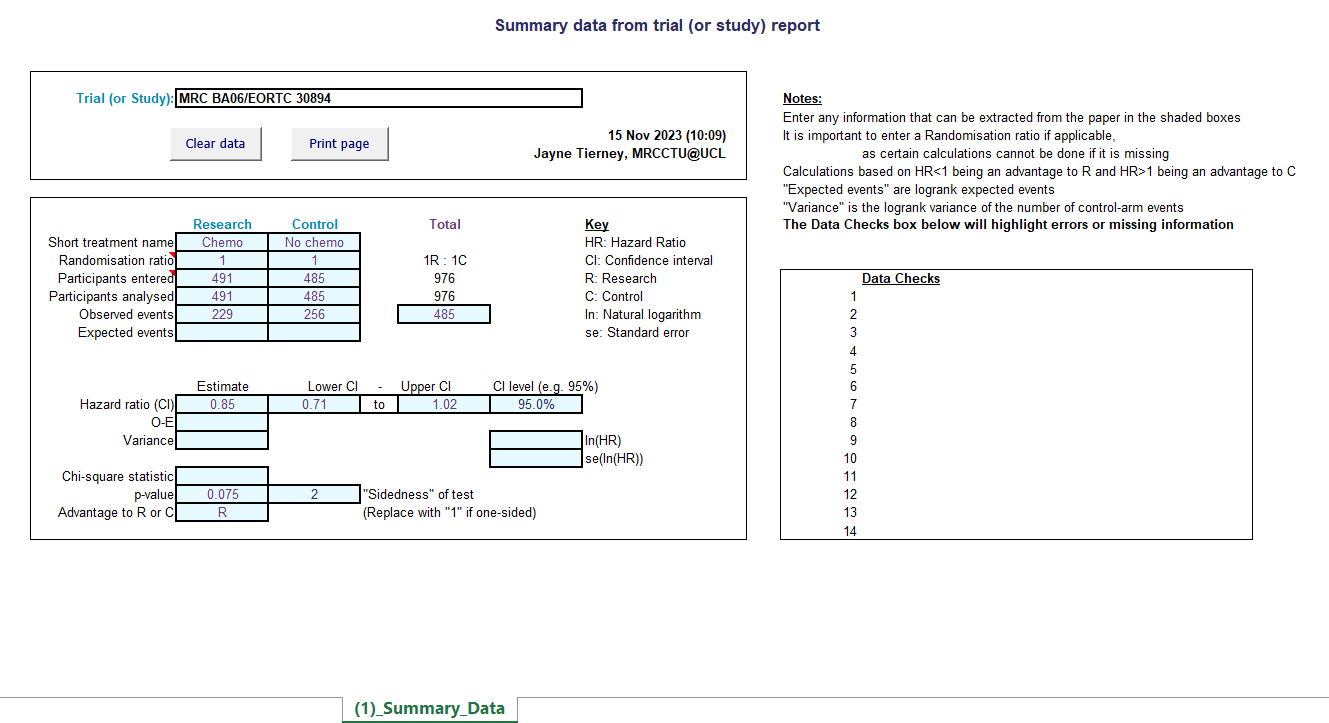


## Figure AF3.3: Calculations spreadsheet: (a) Curve data sheet, (b) Curve copy sheet

(a)


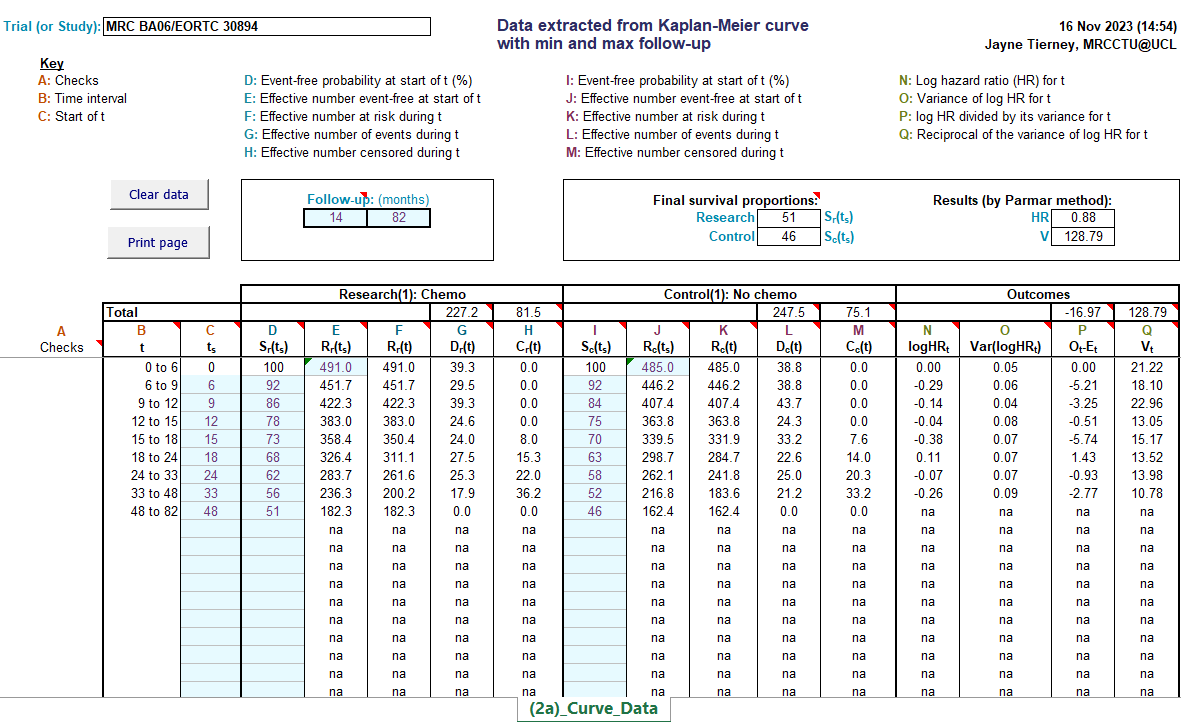


(b)


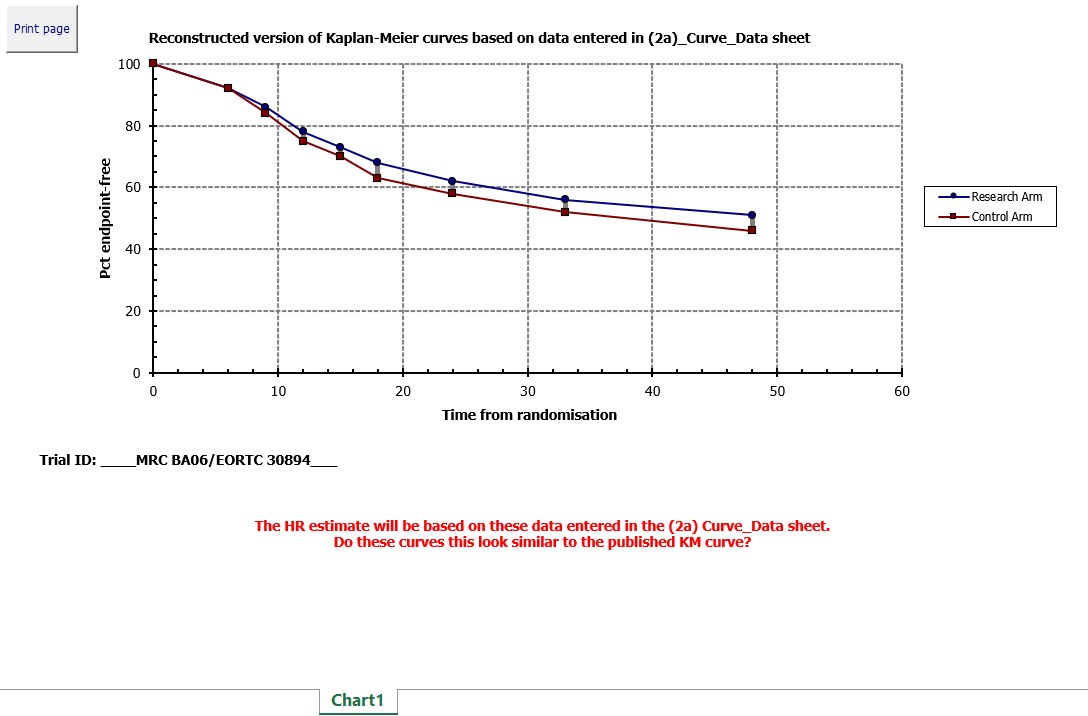


## Figure AF3.4: Calculations Spreadsheet: Output information


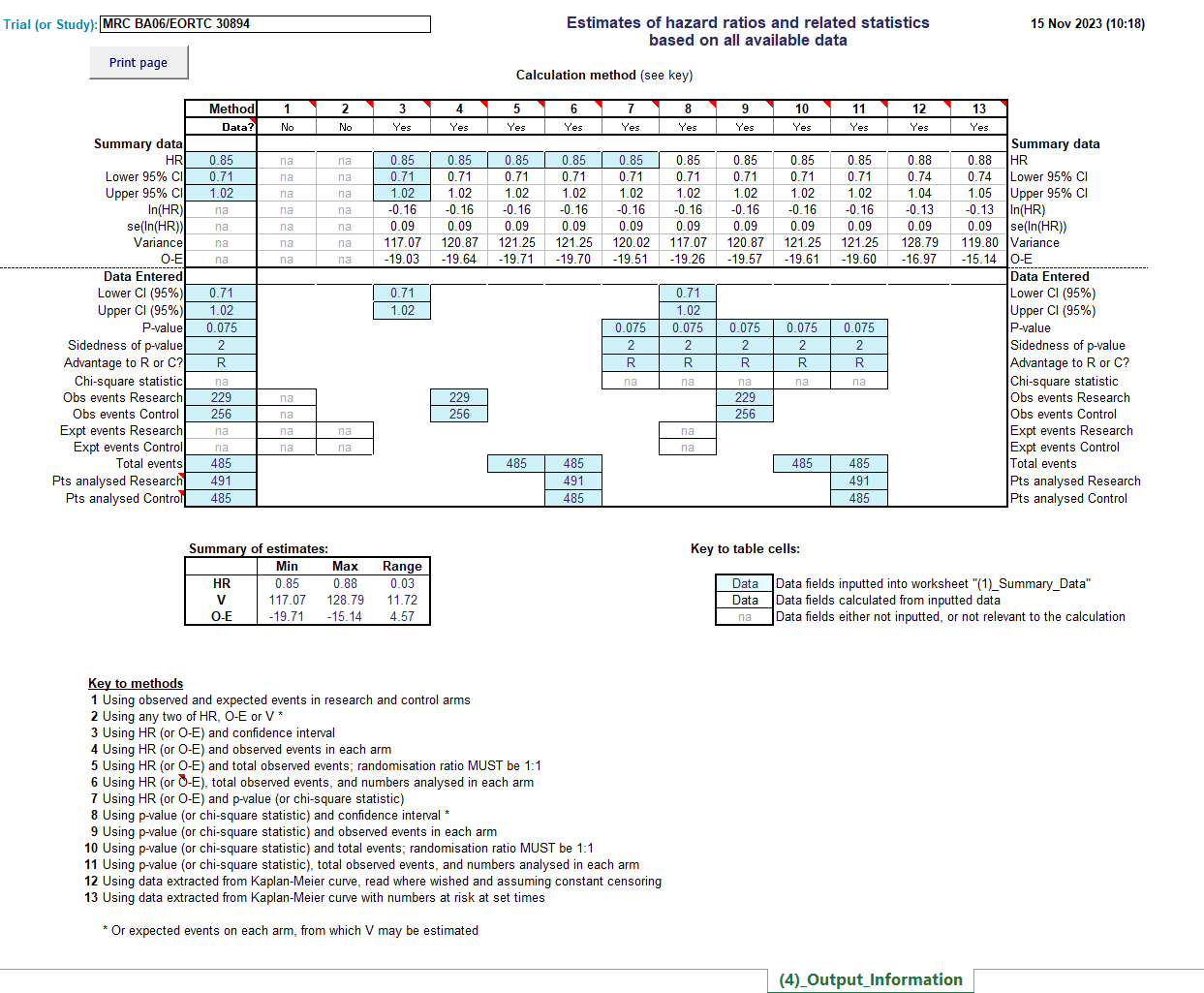

Supplement: Supplementary file 4 — Additional file 4. Spreadsheet guide. [file 13643_2025_2752_MOESM4_ESM.docx]
